# Freezing-Rain Experience and Household Preparedness: A Bayesian Network Analysis of Disruption Severity and Risk Normalization

**DOI:** 10.3390/bs16091533

**Published:** 2026-08-31

**Authors:** Rui Cheng, Xuanyu Zhang, Changqi Dong, Jida Liu

**Affiliations:** 1 School of Management, Harbin Institute of Technology, Harbin 150001, China; chengrui@stu.hit.edu.cn; 2 Department of Aeronautical and Aviation Engineering, The Hong Kong Polytechnic University, Hong Kong SAR 999077, China; xuanyu.zhang@connect.polyu.hk

**Keywords:** freezing rain, household preparedness, disaster experience, risk normalization, conditional associations, Bayesian network

## Abstract

Households may have different preparedness profiles for freezing rain depending on the severity of previous disruptions. This study examines the conditional associations among experience frequency, disruption severity, risk normalization, and household preparedness. Cross-sectional survey data from 533 adult respondents representing individual households in five cities in the Hunan and Hubei Provinces were analyzed using a 12-node Bayesian network integrating disaster experience, risk normalization, risk perception, preparedness knowledge, efficacy beliefs, preparedness constraints, and two preparedness outcomes. Scenario inference, 2000 case-resampling bootstrap replications, repeated stratified 10-fold cross-validation, and parameter sensitivity analysis were conducted. Increasing disruption severity was associated with higher probabilities of risk perception, preparedness knowledge, response efficacy, supplies and outage preparedness, and mobility and care continuity preparedness. Under low disruption severity, repeated experiences corresponded to a 75% probability of high risk normalization, whereas the probabilities of high supplies and outage preparedness and high mobility and care continuity preparedness were 12% and 13%, respectively. Repeated cross-validation showed stronger predictive discrimination for supplies and outage preparedness than for mobility and care continuity preparedness, with mean macro-AUC values of 0.801 and 0.698, respectively; the latter result was interpreted as exploratory. The findings indicate that the preparedness implications of disaster experience depend more on experienced consequences than on exposure frequency alone and support differentiated, calibrated, and context-sensitive preparedness guidance.

## 1. Introduction

Freezing rain is a distinctive winter hazard in which supercooled liquid precipitation freezes on roads, trees, buildings, power lines, and other exposed surfaces. Persistent ice accretion can disrupt transportation, damage electricity and communication infrastructure, restrict access to food and medical services, and expose households to prolonged power and heating interruptions. Unlike ordinary snowfall, freezing rain may require households to remain indoors while simultaneously coping with disrupted electricity, mobility, communication, and essential supplies. The December 2013 ice storm in Ontario, Canada, illustrates these interconnected risks: widespread power outages were associated with increased emergency department visits for injuries, while loss of heating and unsafe alternative heating created additional health threats (Rajaram et al., 2016). Because freezing rain is often episodic, spatially uneven, and difficult for households to assess before substantial ice accumulation occurs, household preparedness, including supplies, outage planning, travel adjustment, and arrangements for vulnerable family members, is an important component of societal safety in exposed areas.

Household preparedness is better understood as a protective decision-making process rather than as the simple possession of emergency supplies (Wilcox et al., 2022). The Protective Action Decision Model links environmental and social cues, risk information, threat perceptions, protective-action perceptions, and situational constraints to decisions about whether and how to act (Lindell & Perry, 2012). Within this framework, risk perception alone may be insufficient for preparedness; households must also know what actions are appropriate, believe those actions can reduce harm, and have the resources and confidence to implement them. A recent systematic review similarly shows that risk perception, response efficacy, self-efficacy, perceived benefits, information, and social conditions can promote or constrain household disaster preparedness (Ni et al., 2025). Freezing-rain preparedness should therefore be examined as the outcome of interconnected cognitive evaluations and practical conditions rather than as a direct response to hazard exposure alone (Kim & Kim, 2022).

Previous disaster experience is often regarded as a source of preparedness learning because direct experience can make hazards personally salient, reveal weaknesses in household arrangements, and provide practical knowledge about needed resources. However, disaster damage may matter more than the mere accumulation of events: Bian et al. (2022) found that damage significantly influenced preparedness behavior, with risk perception mediating this relationship. Reviews of natural-hazard behavior also identify personal experience as an important determinant of risk perception while showing that perceived risk does not consistently translate into precautionary action (Wachinger et al., 2013). Thus, the preparedness implications of freezing-rain experience may depend not only on whether a household has encountered the hazard, but also on the consequences reported and the lessons learned from those consequences (Ng, 2023).

Repeated low-disruption experience may also be associated with a risk-normalization profile: when warnings or hazardous conditions recur without substantial harm, households may infer that future events will be manageable. Research on the normalization of deviance shows that repeated exposure to unsafe conditions without adverse consequences can make such conditions appear increasingly acceptable (Sedlar et al., 2023). In freezing-rain-prone areas, repeated forecasts, temporary road icing, or brief travel disruption may foster familiarity while obscuring the possibility of prolonged outages or serious household losses. Such low-disruption experiences may reduce attention to warnings and weaken the perceived need for advance supplies and outage planning.

These competing possibilities reveal three gaps: first, disaster experience is often measured as a binary condition, without distinguishing exposure frequency from disruption severity; second, preparedness research has emphasized the learning benefits of experience and the risk-perception–action gap, but has paid less attention to repeated low-consequence exposure and risk normalization; third, conventional regression models estimate average associations but are less suited to representing conditional dependencies among experience characteristics, cognitive interpretations, action appraisals, household vulnerabilities, and multiple preparedness outcomes. Bayesian networks can represent interdependent disaster-chain relationships and update outcome probabilities when evidence is introduced (Huang et al., 2023).

At the policy level, household preparedness for freezing rain is also relevant to broader disaster-risk-reduction and sustainable-development agendas. The Sendai Framework emphasizes understanding disaster risk and strengthening preparedness for effective response (UNDRR, 2015); household arrangements for power outages, essential supplies, mobility, and care continuity relate to SDG Targets 11.5, 11.b, and 13.1 on disaster impacts, local risk management, and climate resilience (United Nations, 2015); and the 2024 Asia-Pacific Ministerial Conference on Disaster Risk Reduction further emphasized localized, inclusive, and multi-stakeholder resilience (UNDRR, 2024).

**Figure 1:**
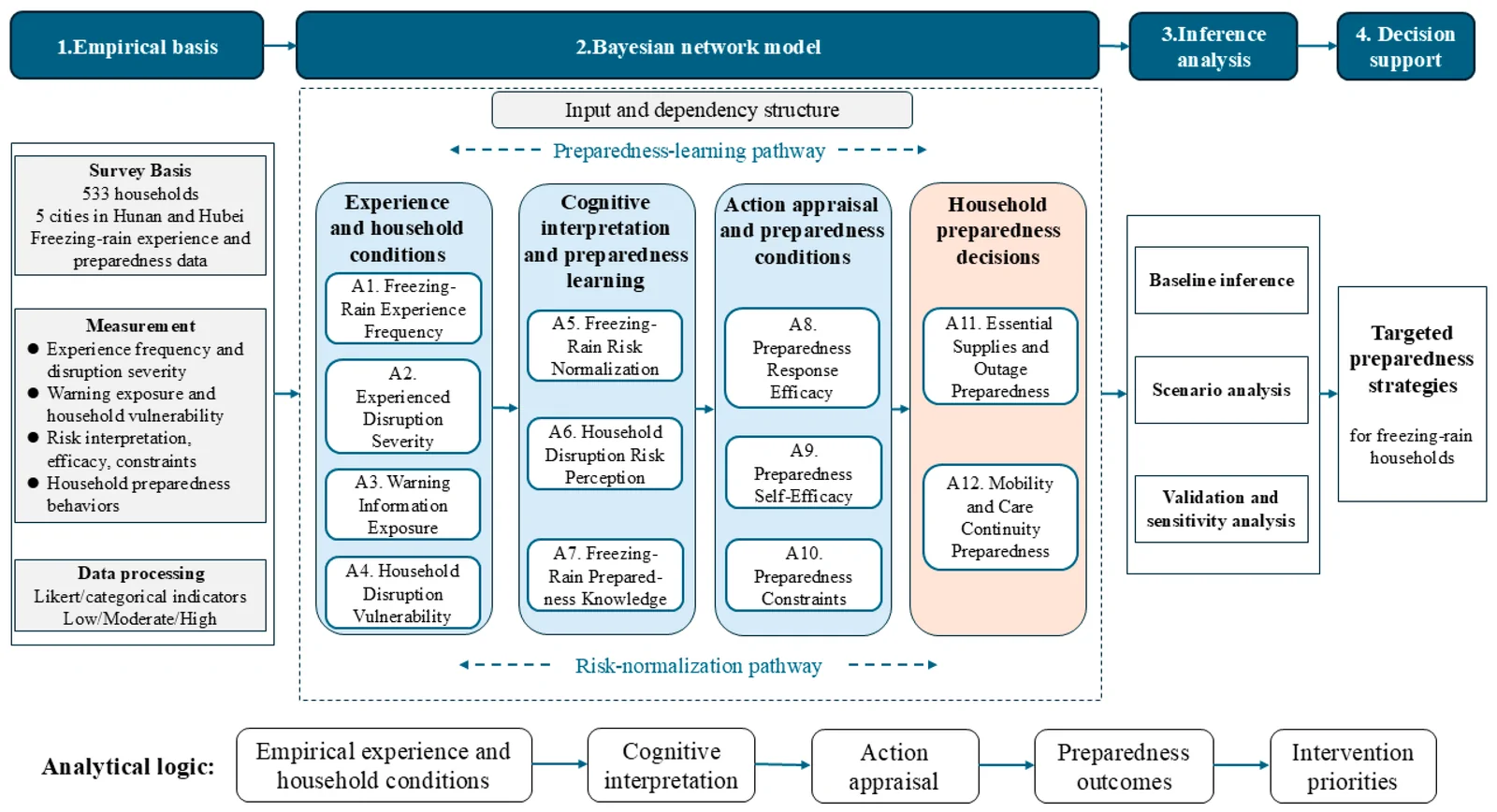
Research framework of the study.

Accordingly, in this study, a Bayesian network model was developed to examine the conditional associations among freezing-rain experience frequency, household disruption severity, risk normalization, cognitive appraisals, and preparedness decisions. This model distinguishes experience frequency from reported disruption severity, compares learning- and normalization-consistent preparedness profiles, and uses scenario-based inference to evaluate household preparedness under different evidence configurations (Figure 1).

## 2. Literature Review

### 2.1. Freezing-Rain Experience and Preparedness Learning

Household preparedness is not an automatic response to hazard exposure but a protective decision-making process shaped by environmental cues, previous experience, and available response options. An empirical application of the Protective Action Decision Model shows that hazard-related preparedness perceptions, stakeholder perceptions, and information interaction mediate the relationship between warning information and protective action (Guo et al., 2022). Population-level evidence from New York State also shows that winter storms, particularly ice storms accompanied by power outages, can increase hospitalizations, comorbidity, and medical costs (Lin et al., 2021). Such consequences may make the need for household supplies, outage plans, and care arrangements more concrete than general awareness of freezing-rain risk.

Previous disaster experience can make hazards personally salient, reveal weaknesses in household arrangements, and provide practical knowledge about needed resources. Social-cognitive approaches view preparedness as a developmental process in which risk awareness must be translated into intentions, capabilities, and protective actions (Paton, 2003). However, awareness alone may be insufficient; evidence from Bucharest indicates that risk perception may not directly influence preparedness behavior or intention (Dobre et al., 2025), and perceived household readiness may diverge from completed preparedness actions (Brown et al., 2021). The relevance of experience for preparedness may therefore depend not only on whether an event occurred, but also on whether its consequences were sufficiently salient to support attention, learning-consistent appraisal, and preparedness-related adjustment.

The severity and meaning of experiences may be more important than exposure frequency alone. Becker et al. (2017) found that prior earthquake experience informed preparedness through the recognition of hazard consequences, observation of effective or ineffective responses, and reflection on future protective actions. Large-scale household evidence similarly shows that experience of disaster damage is positively associated with emergency-supply preparation, although associations differ across basic supplies, energy and heat resources, and evacuation-related preparations (Onuma et al., 2017). Furthermore, flood-hazard research demonstrates that households do not adopt all protective measures as a single package (Grover et al., 2022).

Experience-based learning is more likely to correspond to preparedness when households believe that recommended measures are effective and feasible. Response efficacy and self-efficacy may differ across vulnerable household types (Qiu et al., 2023). Self-efficacy can mediate the relationship between place attachment and preparedness (Wang et al., 2021), and perceived coping efficacy can link household conditions with preparedness behavior (Miao & Zhang, 2023). Severe freezing-rain disruption may therefore be associated with stronger preparedness knowledge and protective action because it reveals both the costs of being unprepared and the practical value of advance arrangements. Accordingly, the following hypothesis is proposed:

**H1.**  *Among households with limited prior freezing-rain experience, greater experienced disruption severity is associated with higher probabilities of supplies and outage preparedness as well as mobility and care continuity preparedness.*

### 2.2. Repeated Experiences and Risk Normalization

Repeated hazard experiences are not necessarily associated with progressively higher preparedness. When an event or warning is followed by little or no personal harm, individuals may use the limited consequences of that experience to revise future risk judgments downward. Mileti and O’Brien (1992) described this as normalization bias in their study of public responses to aftershock warnings after the Loma Prieta earthquake: residents who experienced the initial earthquake without substantial damage tended to interpret the absence of serious consequences as evidence that subsequent threats would also be limited. Normalization is therefore not simply a lack of hazard awareness, because awareness does not necessarily translate into preparedness action (Daimon et al., 2023); rather, it is an experience-based judgment through which a potentially dangerous condition is treated as familiar, manageable, and insufficient to justify additional protection.

Near-miss research offers a related explanation for why repeated low-consequence exposure may weaken preparedness. Near misses are events that could plausibly have caused serious harm but ended without substantial losses; decision-makers may interpret such outcomes as successful experiences and perceive similar future situations as safer. Dillon and Tinsley (2008) found that near-miss information could reduce perceived risk and increase subsequent risk taking: a near miss can support learning when attention is directed toward the disaster that almost occurred, but may foster unwarranted confidence when attention focuses on the favorable outcome. Repeated freezing-rain events involving road icing or brief service disruption without serious household losses may result in the latter.

Weather-warning research points to a similar risk. Barnes et al. (2007) argued that warning outcomes should not be classified only as hits or false alarms because near-threshold events may still produce meaningful local impacts. However, repeated warnings perceived as excessive or unnecessary can reduce confidence in warnings and encourage a “cry wolf” response. Experimental evidence further indicates that high false-alarm rates can impair weather-related decision quality, although explicit uncertainty information may reduce this effect (LeClerc & Joslyn, 2015).

Risk interpretations are also embedded in social and cultural contexts. Although the PADM emphasizes individual threat appraisal and protective decision-making, the Cultural Theory of Risk proposes that shared values and group relations influence which hazards are regarded as salient, tolerable, or manageable (Douglas & Wildavsky, 1982). Repeated low-disruption events may acquire a collectively reinforced meaning of routine manageability, whereas collective memories of severe disruption may sustain concern, and social capital may also affect access to trusted warnings and practical assistance (Aldrich & Meyer, 2015). These perspectives complement, rather than form directly tested components of, the present framework.

Freezing rain is especially relevant because exposure can be recurrent while consequences remain variable. Residents may receive repeated freezing-rain or road-icing warnings and yet experience only minor travel inconvenience, short-lived ice accumulation, or no direct household disruption. Such experience may increase familiarity without revealing the potential for prolonged power loss, heating interruption, restricted access to medicine and food, or disruption of family care. Households may therefore infer that freezing rain is routine rather than a threat requiring advance supplies and continuity planning. Accordingly, the following hypothesis is proposed:

**H2.**  *Under low-disruption conditions, repeated freezing-rain experiences are associated with greater risk normalization and lower household preparedness compared with limited experience.*

### 2.3. Experience Patterns and Household Preparedness

The relationship between disaster experience and household preparedness is unlikely to be uniformly positive or negative. Recent studies indicate that the behavioral meaning of experience depends on the form of exposure, the severity of its consequences, and the conditions under which households interpret and respond to it. In Finland, households that had recently suffered storm-related harm were more likely to adopt preparedness measures than households without such harm, suggesting that experienced consequences may be more influential than general exposure to hazardous weather (Nikkanen et al., 2021). Evidence from hurricane-related purchasing behavior similarly shows that experience of a severe hurricane is associated with stronger subsequent stockpiling than that of a mild hurricane, whereas a previous false alarm can reduce current preparedness (Bilen et al., 2026). Experience frequency and disruption severity should therefore not be treated as interchangeable indicators of hazard exposure.

The relevance of direct experience may also vary across preparedness outcomes and household contexts, including place attachment and the type of preparedness considered (Wallis et al., 2022). Li et al. (2024) found that prior extreme-weather experience shaped general preparedness and evacuation-related decisions through different psychological processes among low-income urban residents: directly affected individuals became more self-reliant in general preparation, while evacuation decisions continued to depend on perceptions of the authorities and other stakeholders. Cisternas et al. (2024) likewise found that household preparedness differed across hazards and cities, even where populations had previously experienced substantial disaster consequences. Greater preparedness for familiar earthquake and tsunami risks than for floods and fires suggests that experience with one hazard does not necessarily correspond to broad or transferable preparedness.

Cognitive and social conditions further shape how experience is interpreted. Trust in and assistance from stakeholders may affect whether awareness is translated into preparedness action (Ao et al., 2022), while cross-national evidence shows that disaster risk perceptions vary across sociocultural settings (Bodas et al., 2022). In a typhoon-prone area, risk perception was associated with preparedness intentions and behavior, but its effects also operated through social and behavioral evaluations (Ng, 2022). A household flood study in Thailand found that preparedness knowledge, perceived risk, and perceived consequences were stronger predictors of preparedness than basic sociodemographic characteristics (Thapa et al., 2025). Prior disaster experience may also correspond to both preparedness and denial or avoidance patterns, depending on resilience and coping dimensions (Wu et al., 2025). These studies indicate that similar exposure histories may be associated with different decisions because households differ in what they experienced, what they inferred, and how they evaluated their capacity to respond.

For freezing rain, four experience patterns are especially relevant. Households with no or limited direct experience may lack personally salient information about prolonged outages and restricted mobility, while limited but severe experiences may correspond to a learning-consistent preparedness profile because a disruptive event reveals the consequences of being unprepared. Repeated low-disruption experiences may instead correspond to a normalization-consistent profile if warnings or minor icing repeatedly occur without substantial losses. In contrast, repeated high-disruption experiences may be associated with stronger preparedness incentives, although behavior may still depend on household resources and vulnerabilities. The preparedness implications of freezing-rain experience should therefore be evaluated through the joint configuration of event frequency and household disruption severity rather than through either dimension alone. Accordingly, the following hypothesis is proposed:

**H3.**  *Household preparedness differs across combinations of freezing-rain frequency and disruption severity.*

## 3. Materials and Methods

This section describes the study design, data collection, variable measurement, and construction of the theory-driven Bayesian network, and also outlines the procedures used to evaluate model performance, conduct scenario inference, and assess parameter sensitivity.

### 3.1. Study Design and Data Collection

A cross-sectional questionnaire was used to examine the relationships among freezing-rain experience, risk normalization, and household preparedness. The survey was conducted in five freezing-rain-prone cities in central China, purposively selected to capture variation in freezing-rain exposure, infrastructure disruption, climatic conditions, and household preparedness contexts: Changsha, Yueyang, and Huaihua in Hunan Province, and Wuhan and Yichang in Hubei Province. Recruitment was organized by city and used non-probability convenience sampling within each city; therefore, “stratified” refers to recruitment organization across the five cities rather than probability-based stratified sampling. Participants were recruited from residential communities with assistance from local community staff and through online invitations circulated within local community networks. The household was treated as the unit of analysis, with one eligible adult respondent reporting for each participating household. A1 recorded the number of freezing-rain events that respondents regarded as directly affecting their household, whereas A2 separately recorded the maximum reported incidences of freezing-rain-related disruption to mobility, services, health, or property; thus, the latter was not deterministically assigned Low when A1 was None.

Eligible participants were at least 18 years old, had lived in the study city for at least three consecutive years, and were familiar with their household’s emergency supplies, outage arrangements, travel decisions, and care responsibilities. Only one respondent per household was permitted. The questionnaire was developed from previous research on disaster experience, protective decision-making, risk normalization, efficacy appraisal, and household preparedness. Eight experts in disaster management, behavioral science, meteorology, and community emergency preparedness reviewed the initial questionnaire, and a pilot survey with 45 eligible adults assessed item clarity, completion time, response distributions, and preliminary reliability. Pilot participants were excluded from the final sample, and unclear, repetitive, or contextually inappropriate items were revised before formal data collection.

Formal data collection was conducted from May 2025 to April 2026 using identical online and community-based questionnaires. Participants received the study information, gave informed consent, completed eligibility screening, and provided anonymized responses; no financial or material incentives were offered. A total of 610 questionnaires were received, of which 77 were excluded because of duplicate submissions, implausibly short completion times, invariant response patterns, or logical inconsistencies. The final analytical sample included 533 responses, corresponding to a data-retention rate of 87.4%. A conventional response rate could not be calculated because the number of individuals reached through the recruitment channels was not recorded. The achieved sample included 148 households from Changsha, 107 from Yueyang, 66 from Huaihua, 130 from Wuhan, and 82 from Yichang.

### 3.2. Measures and Node Definition

The questionnaire combined adapted measures with hazard-specific item development. Warning information exposure (A3), household disruption risk perception (A6), preparedness response efficacy (A8), preparedness self-efficacy (A9), and preparedness constraints (A10) were adapted from established disaster-preparedness frameworks and instruments, including the Protective Action Decision Model, the General Disaster Preparedness Belief Scale, protection-motivation-based household preparedness measures, and national household preparedness surveys (Grothmann & Reusswig, 2006; Inal et al., 2018; Chen & Cong, 2022). Perceived freezing-rain preparedness knowledge (A7) was developed with reference to household preparedness knowledge and planning measures (Thomas et al., 2015). The two behavioral outcomes, essential supplies and outage preparedness (A11) and mobility and care continuity preparedness (A12), were action checklists informed by household and disaster-specific preparedness measures (Heagele et al., 2020; Osman et al., 2024). Because no established household-level scale was identified for risk normalization after recurrent low-impact freezing rain, the five risk normalization items (A5) were developed from near-miss and risk-normalization research (Dillon et al., 2014; Tinsley et al., 2012). All items were contextualized to freezing-rain-related disruptions and reviewed before pilot testing.

The Bayesian network comprised 12 nodes in four layers: experience and household conditions, cognitive interpretation, action appraisal, and preparedness decisions. Factual variables used categorical or event-based indicators, reflective constructs used five-point Likert scales, and preparedness outcomes used completed-action checklists. Table 1 summarizes the node measurement and state classification.

The first layer captured prior freezing-rain experience and household context. Freezing-rain experience frequency (A1) recorded the number of events the respondents identified as directly affecting their household during the previous five years, while experienced disruption severity (A2) was independently defined as the highest reported severity across transportation disruption, power outage, heating interruption, restricted access to essential supplies, and health or property impacts. Low indicated no consequential disruption or minor short-lived inconvenience; Moderate indicated substantial but temporary disruption without serious health or property consequences; and High indicated prolonged or multiple service disruptions, or serious health or property impacts. Because A1 captured event identification and frequency whereas A2 captured reported consequences, the latter was not deterministically assigned Low when the former was None, and all observed A1 × A2 configurations were retained. Warning information exposure (A3) included four items, and household disruption vulnerability (A4) summed six conditions: older adults, young children, chronic illness, dependence on electrically powered medical equipment, limited transportation access, and limited external assistance.

The second layer represented cognitive interpretation: freezing-rain risk normalization (A5) included five items assessing whether repeated low-impact events made future freezing rain seem familiar, manageable, or unlikely to cause serious consequences; household disruption risk perception (A6) and freezing-rain preparedness knowledge (A7) each included five items.

The third layer represented action appraisal and preparedness conditions: preparedness response efficacy (A8) and preparedness self-efficacy (A9) included four items each, while preparedness constraints (A10) included five items covering financial, spatial, time, housing, and caregiving barriers.

The final layer contained two behavioral outcomes—essential supplies and outage preparedness (a11), and mobility and care continuity preparedness (A12)—measured with a ten-item and eight-item checklist, respectively.

Before constructing the discrete Bayesian network nodes, the seven reflective constructs were evaluated using the 32 original five-point Likert items. A confirmatory factor analysis specified Warning Information Exposure, Risk Normalization, Risk Perception, Preparedness Knowledge, Response Efficacy, Self-Efficacy, and Preparedness Constraints as seven correlated latent factors. Maximum-likelihood estimation was used because the item distributions showed no severe univariate non-normality. Model fit was evaluated using χ^2^, χ^2^/df, CFI, TLI, RMSEA with its 90% confidence interval, and SRMR; internal consistency and convergent validity were assessed using standardized factor loadings, Cronbach’s alpha, composite reliability, and AVE; and discriminant validity was examined using HTMT, with 5000 nonparametric bootstrap samples used to estimate 95% confidence intervals. To assess whether risk normalization was empirically distinct, the seven-factor model was compared with models combining risk normalization with risk perception, combining risk normalization with response efficacy, and loading all 32 items onto one factor. Supplementary Tables S1–S4 report item wording, loadings, model comparisons, and discriminant-validity results.

For Bayesian network analysis, prespecified rules classified variables into three states: for A3 and A5–A10, mean Likert scores were classified as Low = 1.00–2.33, Moderate = 2.34–3.67, and High = 3.68–5.00; for A4, the six binary conditions were summed as Low = 0–1, Moderate = 2–3, and High = 4–6. These thresholds were based on theoretical score ranges and emphasized interpretability rather than data-driven optimization. Robustness was examined using empirical tertile cut points for A3–A12 and stricter high-state definitions, while A1–A2, the network structure, and the CPT estimator remained fixed.

### 3.3. Bayesian Network Construction

A Bayesian network was used to represent the probabilistic dependencies among freezing-rain experience, cognitive appraisal, preparedness conditions, and household preparedness decisions. It consisted of a directed acyclic graph in which each node represented one of the 12 variables defined in Table 1 and each directed arc represented a conditional dependency between two nodes. For a set of variables $X_{1},\ldots ,X_{n}$, the joint probability distribution was factorized as follows:

(1) $$P\left(X_{1},\ldots X_{n}\right)=\prod_{i=1}^{n}P(X_{i}|Pa(X_{i}))$$

where $Pa(X_{i})$ denotes the parent nodes of $X_{i}$. This structure enabled the posterior probabilities of household preparedness outcomes to be updated when evidence concerning freezing-rain frequency, disruption severity, risk normalization, or other conditions was entered into the model.

The network structure was specified on the basis of the four-layer conceptual framework, the Protective Action Decision Model, and the three hypotheses developed in the literature review. Freezing rain experience and household condition nodes were treated as upstream variables: risk normalization, risk perception, preparedness knowledge, response efficacy, self-efficacy, and preparedness constraints were positioned as intermediate variables, while supplies and outage preparedness and mobility and care continuity preparedness were treated as downstream outcome nodes.

**Figure 2:**
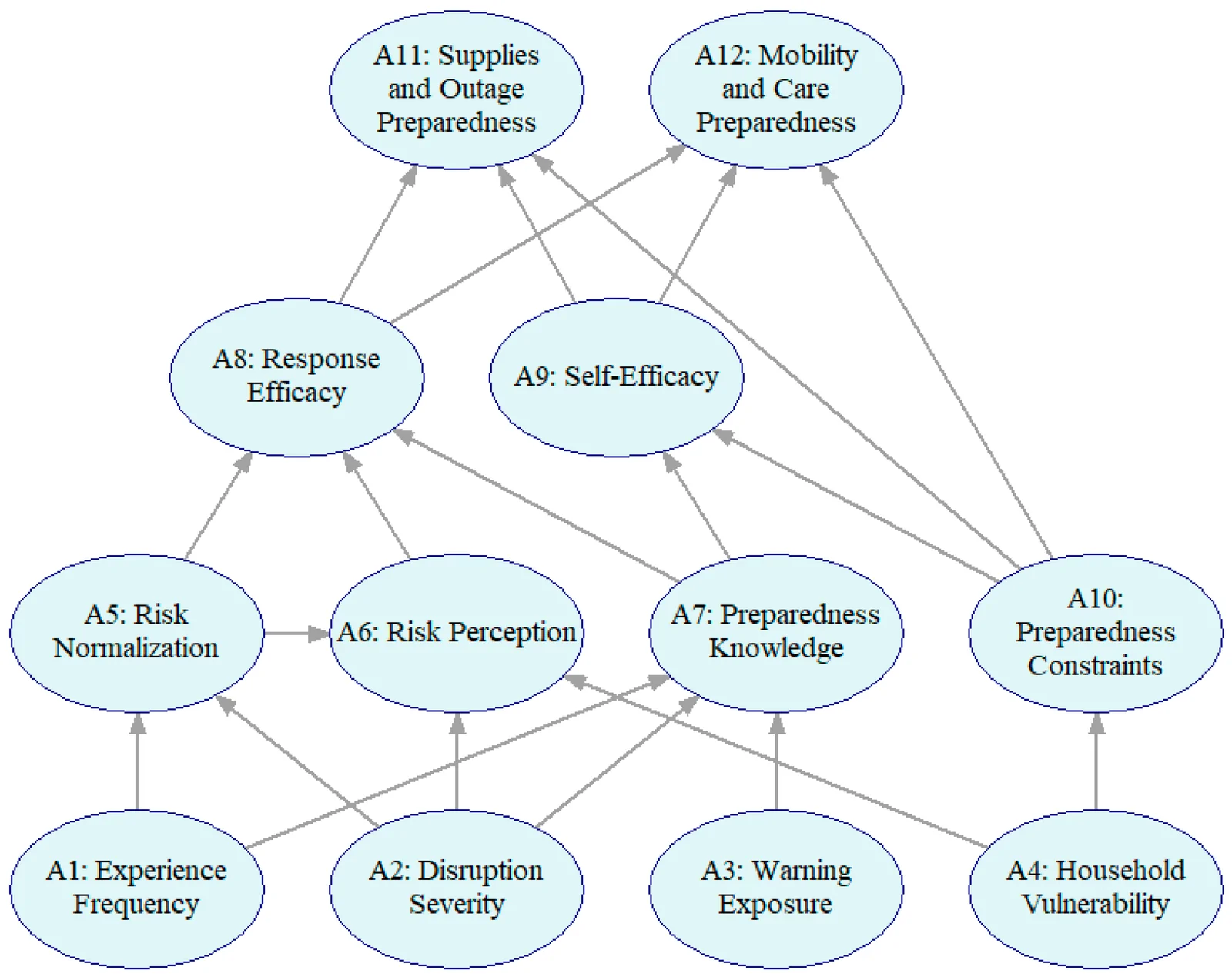
Structure of the theory-driven Bayesian network.

Directed arcs were specified only when theoretically consistent with the proposed conditional relationships among experience, cognitive appraisal, action appraisal, and preparedness outcomes. The arrows indicate conditional dependencies for probability factorization and should not be interpreted as causal or temporal effects. The final theory-driven Bayesian network included 12 nodes and 20 directed arcs, as shown in Figure 2.

CPT coverage was assessed across all 160 parent-state rows by recording zero-frequency rows, rows with *n* < 5, and rows with *n* < 10. The primary model used symmetric Dirichlet smoothing with α = 1/3 per child state, equivalent to a total prior sample size of 1 per CPT row. An unsmoothed maximum-likelihood model was retained for robustness. Coverage diagnostics were implemented in Python 3.13, while GeNIe Modeler 4.0 was used for visualization and sensitivity analysis.

### 3.4. Model Validation and Scenario Inference

Predictive performance was evaluated using 10 repetitions of stratified 10-fold cross-validation, producing 100 test folds for each outcome; in each fold, the network structure was fixed, and the CPTs were re-estimated from the training data. A8–A10 served as categorical predictors for both outcomes. The Bayesian network was compared with a training-fold majority-class predictor and multinomial logistic regression using the same predictors. Accuracy, balanced accuracy, macro-F1, state-specific one-versus-rest AUC, and macro-AUC were summarized as means and standard deviations across folds.

Scenario inference was conducted by entering evidence into experience frequency (A1) and experienced disruption severity (A2) while leaving the remaining nodes unobserved. H1 was assessed conditionally by fixing experience frequency at Limited and varying disruption severity from Low to Moderate and High, while H2 was assessed by fixing disruption severity at Low and comparing the Limited–Low and Repeated–Low scenarios; the None–Low scenario was retained as a descriptive reference but was not treated as the principal comparison for H2. H3 was assessed by comparing the posterior profiles produced by the Limited–Low, Limited–High, Repeated–Low, and Repeated–High configurations. The corresponding scenarios and assessment criteria are summarized in Table 2.

Uncertainty in the principal scenario contrasts was quantified using 2000 nonparametric case-resampling bootstrap samples. In each replicate, 533 cases were sampled with replacement, while the 20-arc structure, discretization thresholds, and primary symmetric Dirichlet estimator (α = 1/3 per child state) were held fixed. All CPTs and scenario posteriors were re-estimated, and bootstrap means, standard errors, and percentile 95% confidence intervals were calculated for the posterior probability differences.

One-way parameter sensitivity analyses for A11 = High and A12 = High were conducted in GeNIe Modeler 4.0 under the baseline network without evidence, using a 10% parameter spread. Each CPT parameter was varied separately, while GeNIe proportionally adjusted the other probabilities in the same row to preserve a row sum of 1. Parameters were ranked by the resulting target-posterior probability range, and the ten largest ranges were displayed in tornado diagrams.

## 4. Results

This section presents the sample characteristics and measurement assessment, baseline Bayesian network probabilities, scenario-inference results, and model validation and sensitivity analyses.

### 4.1. Sample Characteristics and Measurement Assessment

The analytical sample comprised 533 respondents from five cities in the Hunan and Hubei Provinces. Table 3 summarizes the geographical distribution, demographic characteristics, socioeconomic conditions, housing status, and household vulnerability characteristics of the respondents.

As shown in Table 3, 60.2% of respondents were from Hunan and 39.8% from Hubei, women accounted for 52.5%, most respondents were aged 18–49, more than half held at least a bachelor’s degree, income was concentrated below RMB 10,000, and 70.7% lived in owner-occupied housing. The sample also included households with vulnerable members or limited transport/external assistance.

Before discretization, the seven reflective constructs were assessed using 32 Likert items, and the seven-factor CFA showed excellent fit: χ^2^(443) = 415.478, χ^2^/df = 0.938, CFI = 1.000, TLI = 1.003, RMSEA = 0.000, and SRMR = 0.026. Loadings ranged from 0.724 to 0.862.

As shown in Table 4, standardized loadings ranged from 0.724 to 0.862, thus all exceeding 0.70. Cronbach’s alpha and CR ranged from 0.824 to 0.929, and AVE ranged from 0.539 to 0.724, supporting internal consistency and convergent validity. The newly developed freezing-rain risk-normalization scale showed loadings of 0.755–0.802, α = 0.883, CR = 0.883, and AVE = 0.602.

The seven-factor model also outperformed competing models that combined risk normalization with risk perception, combined risk normalization with response efficacy, or specified a single general factor; complete results are reported in Supplementary Table S3. Discriminant validity was further supported by HTMT: all 21 values were below 0.85, with the largest value observed between risk normalization and risk perception (HTMT = 0.679; 95% CI: 0.618–0.731). Supplementary Table S4 reports the complete HTMT results.

### 4.2. Bayesian Network Structure and Baseline Probabilities

The theory-driven Bayesian network comprised 12 nodes and 20 directed links organized into four layers: freezing-rain experience and household conditions, cognitive interpretation, action appraisal, and household preparedness outcomes. As shown in Figure 2, A1–A4 were specified as upstream nodes, A5–A10 as intermediate nodes, and A11–A12 as outcome nodes. A1 and A2 were retained as separate roots because they measured event identification/frequency and reported consequences, respectively. Their observed association was nonsignificant and small—χ^2^(4) = 2.852, *p* = 0.583, and Cramér’s V = 0.052; the full cross-tabulation is reported in Supplementary Table S5. Treating them as separate roots indicates the absence of a direct A1→A2 arc in the specified factorization, not conceptual unrelatedness.

The CPT audit identified seven zero-frequency rows among 160 parent-state rows; 27 rows had N < 5 and 43 had N < 10, mainly in A6, A8, A11, and A12. Node-level diagnostics are reported in Supplementary Table S6, Panel A.

**Figure 3:**
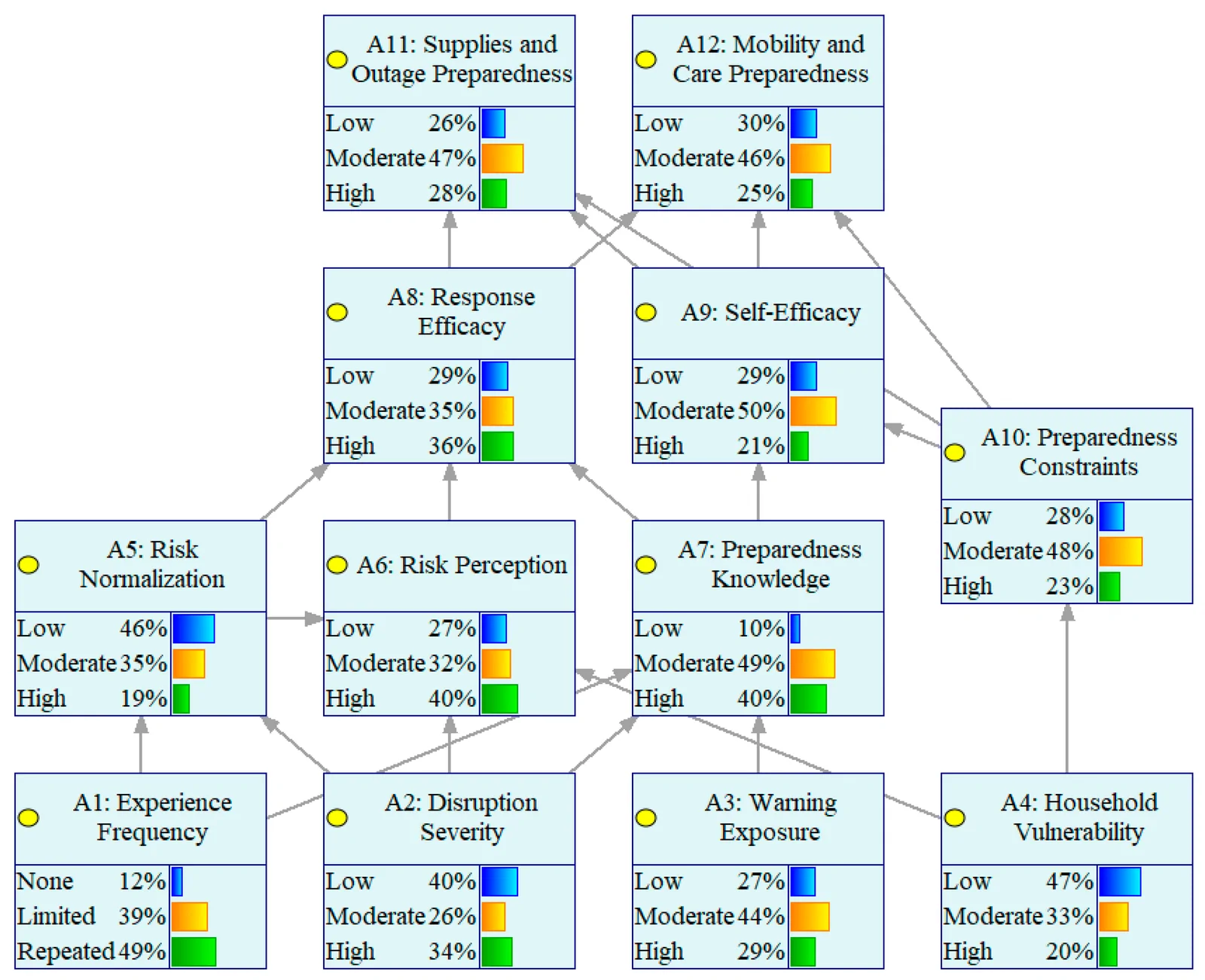
Baseline marginal probabilities in the Bayesian network.

Figure 3 presents the baseline marginal probabilities from the primary symmetric Dirichlet model (α = 1/3 per child state). Repeated experience was the largest A1 state (49%), followed by limited experience (39%) and no directly identified experience (12%). The Low, Moderate, and High A2 states accounted for 40%, 26%, and 34%, respectively. Risk normalization was mostly Low or Moderate, while 40% of households showed High disruption risk perception. Preparedness knowledge was concentrated in the Moderate and High states, response efficacy was relatively even, and self-efficacy and constraints were mainly Moderate. For A11 and A12, Moderate preparedness was most common, while High preparedness accounted for 28% and 25%, respectively.

### 4.3. Scenario Inference

Scenario inference was conducted by entering evidence into the experience-frequency and disruption-severity nodes and observing the resulting changes in the posterior probabilities of the cognitive, action-appraisal, and preparedness nodes. All other nodes were left unobserved so that their probabilities could be updated through the conditional dependencies represented in the Bayesian network. Table 5 reports the posterior probabilities of the principal nodes being in their High states under six combinations of experience frequency and disruption severity. These results provide an overall comparison of the cognitive and preparedness profiles associated with different freezing-rain experience patterns.

**Figure 4:**
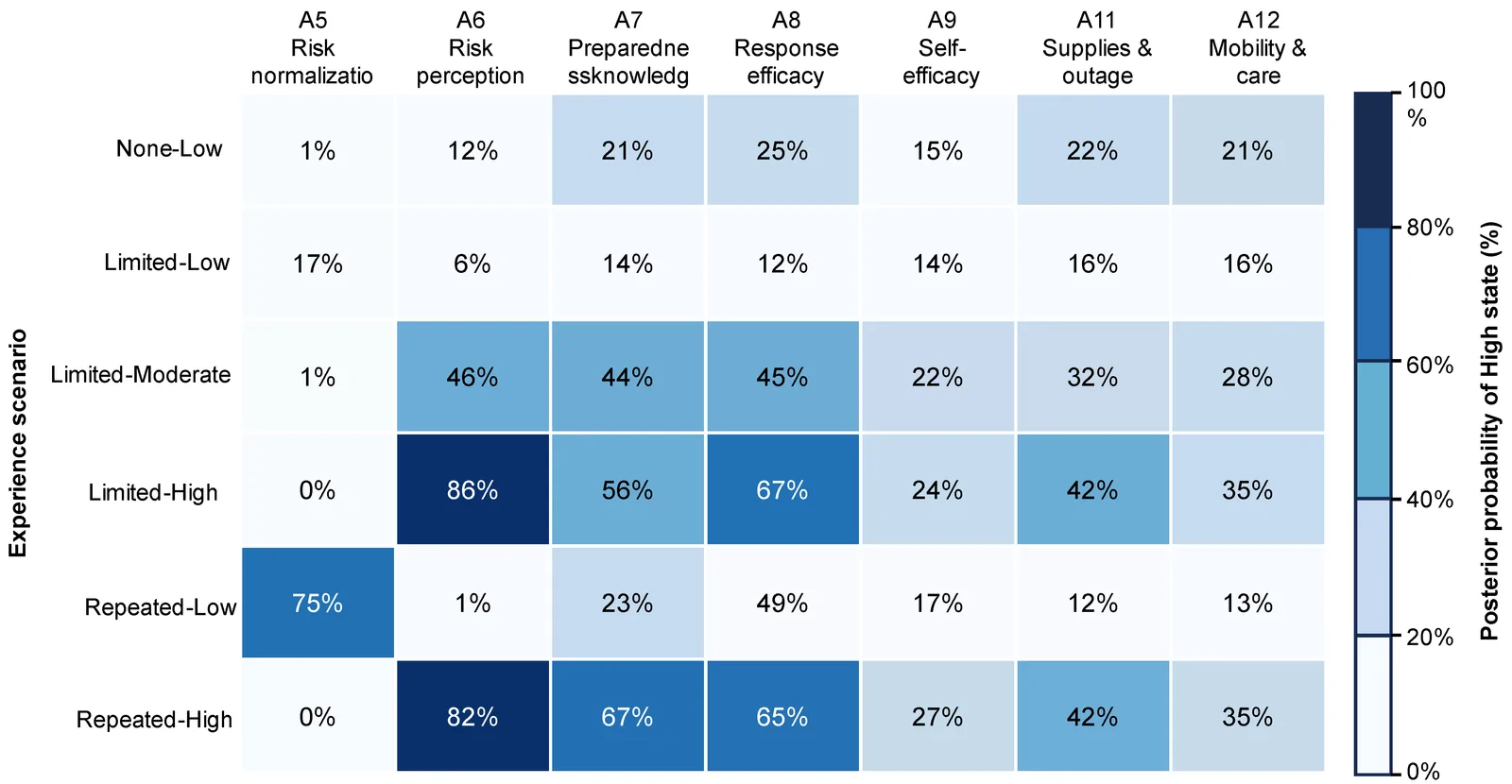
Heatmap of posterior probabilities across Bayesian scenarios.

The results revealed substantial variation across the six scenarios, as shown in Figure 4.

Under the Limited–Low scenario, the posterior probabilities of high risk perception, preparedness knowledge, response efficacy, supplies and outage preparedness, and mobility and care continuity preparedness were Low, at 6%, 14%, 12%, 16%, and 16%, respectively. When disruption severity increased while experience frequency remained Limited, these probabilities rose markedly. Under Limited–High, high risk perception, preparedness knowledge, and response efficacy reached 86%, 56%, and 67%, while high supplies and outage preparedness and high mobility and care continuity preparedness reached 42% and 35%, respectively.

A contrasting pattern appeared under repeated low-disruption experiences. Repeated–Low corresponded to a 75% probability of high risk normalization, while high risk perception and response efficacy declined to 1% and 4%, and the two high-preparedness probabilities were only 12% and 13%. Under Repeated–High, risk normalization fell to approximately zero, whereas risk perception, preparedness knowledge, response efficacy, and both preparedness outcomes increased substantially. These results suggest that the behavioral meaning of repeated experiences depends strongly on disruption severity.

**Figure 5:**
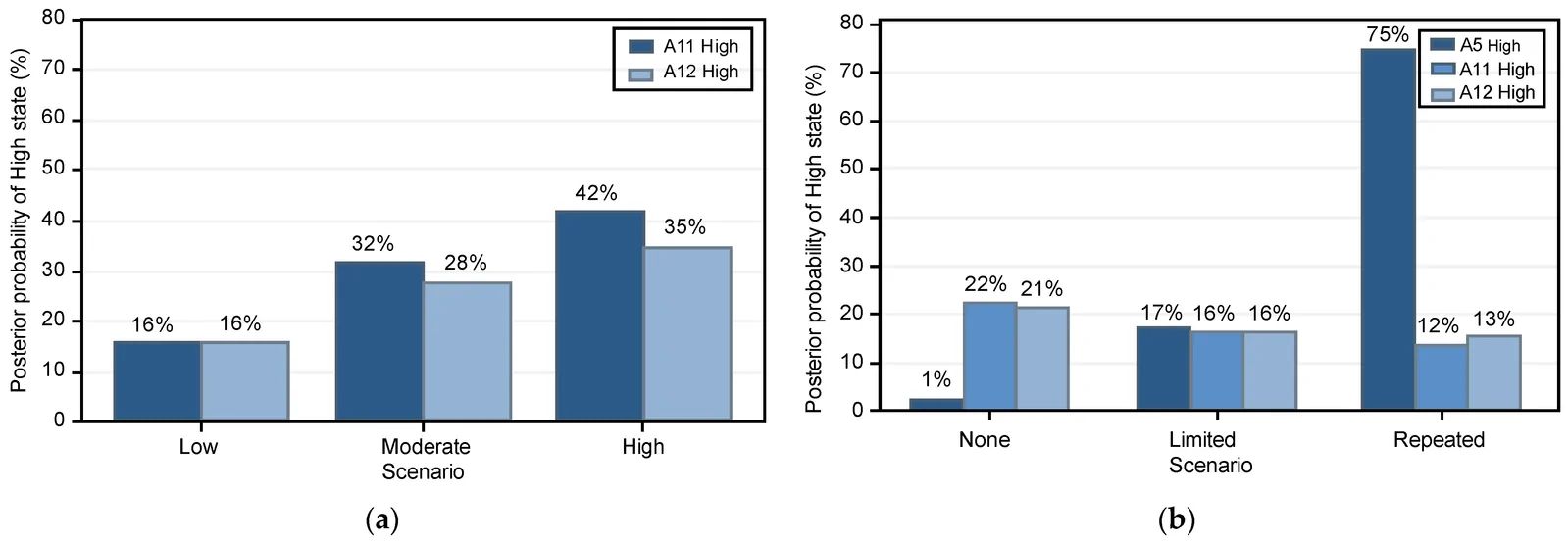
Posterior probability changes under the Bayesian scenarios. (**a**) Preparedness probabilities by disruption severity (Limited experience); (**b**) Risk-normalization and preparedness probabilities by experience frequency (Low disruption).

To provide a clearer visual comparison of the evidence relevant to H1 and H2, Figure 5 presents the principal posterior probability changes.

As shown in Figure 5a, increasing disruption severity was associated with a monotonic rise in both preparedness outcomes when experience frequency was fixed at Limited: high A11 increased from 16% to 32% and 42%, while high A12 increased from 16% to 28% and 35%. Table 5 further shows that higher severity was accompanied by stronger risk perception, preparedness knowledge, and response efficacy, while risk normalization declined. Bootstrap contrasts in Table 6 were used to evaluate uncertainty.

Figure 5b presents posterior changes under low severity. The formal H2 comparison was Limited–Low versus Repeated–Low, with None–Low retained as a descriptive reference. High risk normalization rose from 1% to 17% and 75%, whereas high A11 declined from 22% to 16% and 12%, and high A12 from 21% to 16% and 13%; therefore, the dominant change was increased risk normalization rather than a large preparedness decline. Repeated low-disruption experiences were not accompanied by a cumulative-learning-consistent increase in preparedness; the preparedness differences were modest relative to the normalization increase.

**Figure 6:**
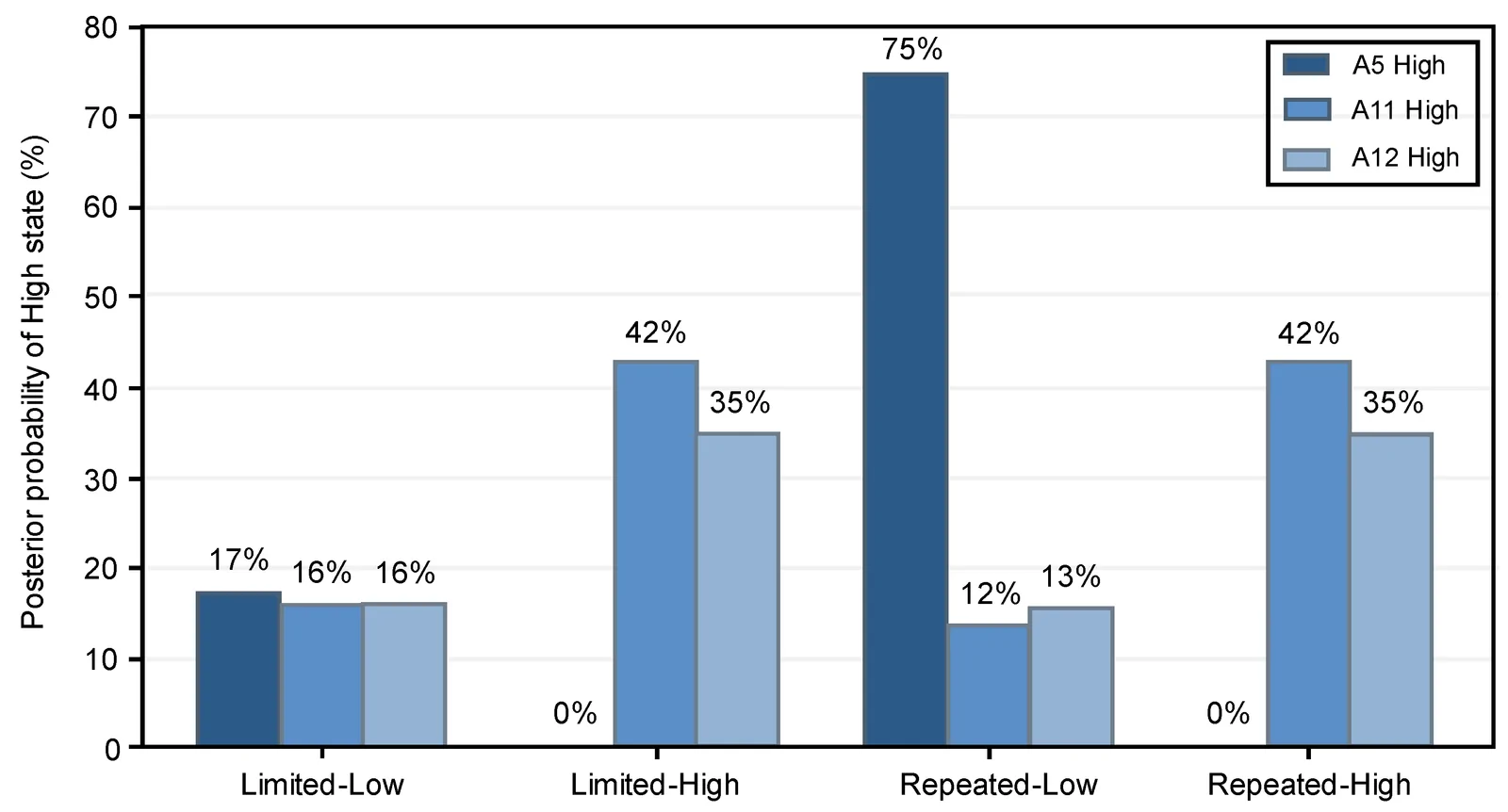
Posterior probabilities under the four joint experience configurations.

Figure 6 further demonstrates that experience frequency and disruption severity showed joint conditional associations with the posterior profiles.

The Repeated–Low configuration showed the highest probability of risk normalization and the lowest probabilities of both high-preparedness outcomes. In contrast, both high-severity scenarios showed near-zero high risk normalization and substantially higher preparedness probabilities. For both Limited–High and Repeated–High, high supplies and outage preparedness reached 42%, while high mobility and care continuity preparedness reached 35%.

Differences between Limited–High and Repeated–High remained visible in intermediate nodes: Repeated–High corresponded to higher preparedness knowledge than Limited–High, increasing from 56% to 67%, and to slightly higher self-efficacy, increasing from 24% to 27%. However, these differences were not accompanied by further increases in the two preparedness outcomes; thus, high disruption severity showed a stronger association with outcome probabilities than experience frequency. Repeated experiences were associated with high risk normalization mainly under low-disruption conditions, whereas high-disruption configurations were associated with higher risk recognition, preparedness knowledge, and preparedness probabilities. These conditional patterns were consistent with H3, although the difference-in-differences estimates were modest; the corresponding estimates and bootstrap confidence intervals are reported in Table 6.

To quantify sampling uncertainty, Table 6 reports the bootstrap distributions of the principal posterior probability contrasts used to assess H1–H3.

All stepwise H1 contrasts were positive, with their 95% confidence intervals excluding zero. For H2, Repeated–Low versus Limited–Low increased the probability of high risk normalization by 58.4 percentage points (95% CI: 47.2–70.0) and reduced the probabilities of high A11 and A12 preparedness by 4.0 and 3.1 percentage points, respectively. The H3 difference-in-differences was 3.7 and 3.2 percentage points for A11 and A12, respectively. Accordingly, H1–H3 were supported, although the H2 preparedness reductions and H3 interaction contrasts were modest.

The alternative discretization rules produced the same qualitative patterns (Supplementary Table S6, Panel B): all H1 contrasts remained positive, Repeated–Low remained highest on A5 and lowest on A11 and A12, and both H3 interaction contrasts remained positive, although their magnitudes were attenuated. High-severity scenarios also continued to rank highest on both preparedness outcomes. Thus, the positive direction of the H3 contrasts was preserved across the alternative discretization rules, but their attenuated magnitudes reinforce a cautious interpretation of H3 as a modest conditional interaction pattern.

### 4.4. Model Validation and Sensitivity Analysis

Predictive performance was reassessed using 10 repetitions of stratified 10-fold cross-validation and compared with majority-class and multinomial logistic benchmarks. The results are reported in Table 7.

For A11, the Bayesian network achieved a mean balanced accuracy of 0.650, macro-F1 of 0.648, and macro-AUC of 0.801; these values substantially exceeded the majority-class benchmark and were similar to those of multinomial logistic regression. Predictive performance was weaker for A12, for which the Bayesian network achieved a balanced accuracy of 0.480, macro-F1 of 0.486, and macro-AUC of 0.698. Although these values exceeded the majority-class benchmark, they were lower than the corresponding logistic-regression results; therefore, A12 demonstrated limited-to-moderate predictive discrimination and substantially greater uncertainty than A11.

**Figure 7:**
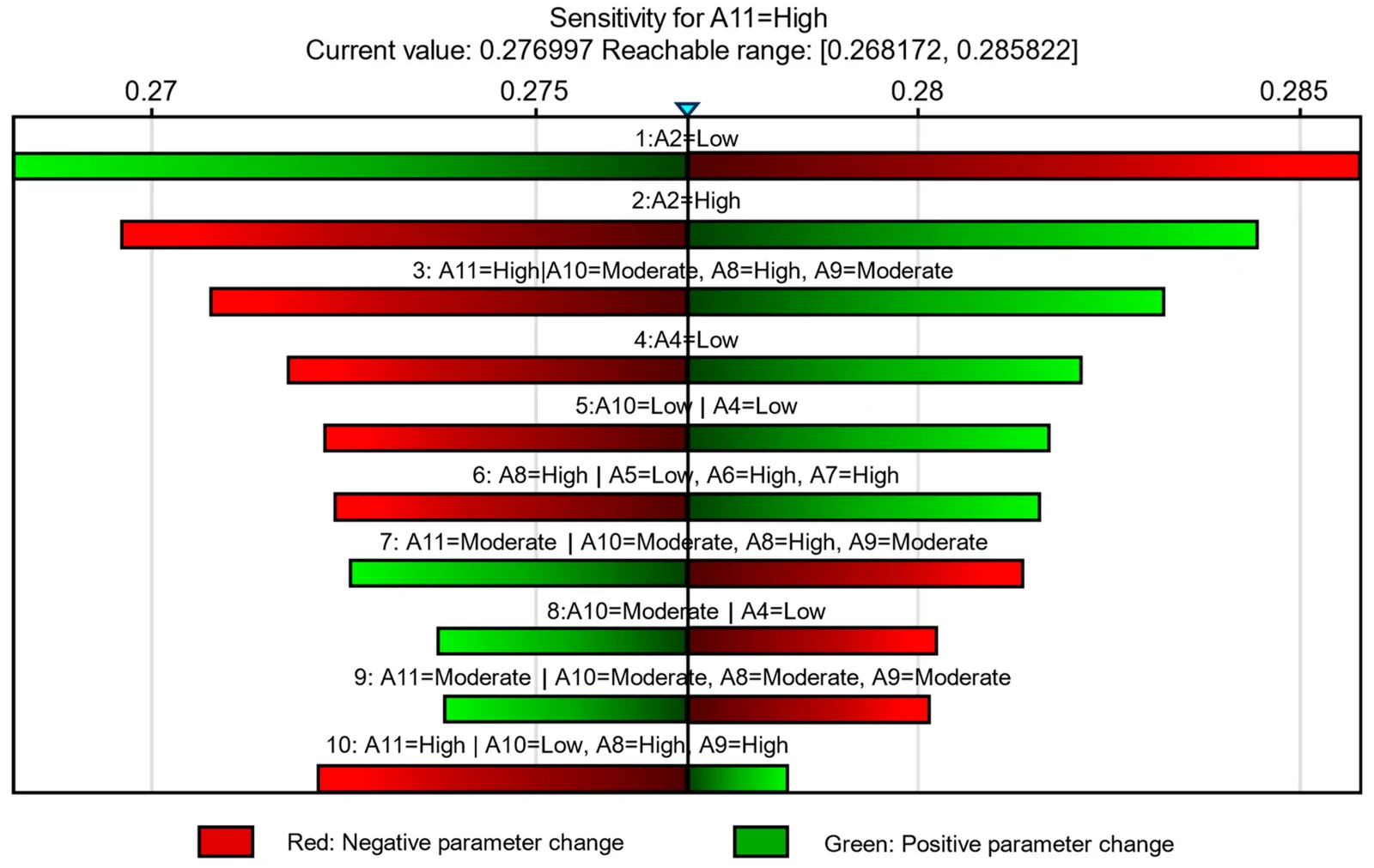
Parameter sensitivity for A11 = High. Parameters were evaluated using a 10% parameter spread in the baseline network without evidence and ranked by the resulting target-posterior probability range.

**Figure 8:**
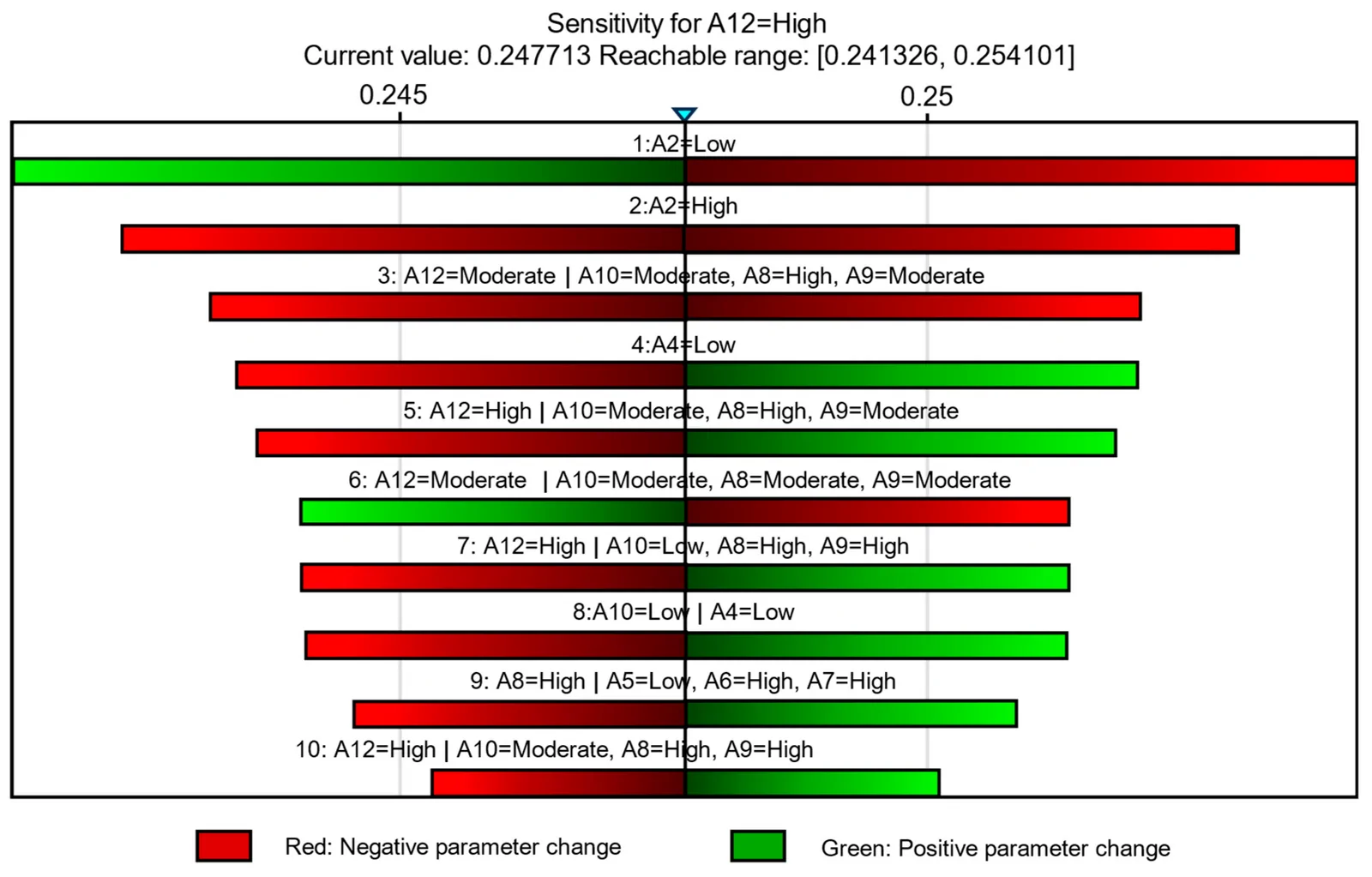
Parameter sensitivity for A12 = High. Parameters were evaluated using a 10% parameter spread in the baseline network without evidence and ranked by the resulting target-posterior probability range.

Figure 7 and Figure 8 present the ten CPT parameters producing the largest target-posterior probability ranges for A11 = High and A12 = High, respectively, under the baseline network without evidence.

The sensitivity profiles of the two outcomes were broadly similar. In both tornado diagrams, CPT parameters involving the Low and High states of experienced disruption severity (A2) produced the widest target-posterior probability ranges. Parameters involving household disruption vulnerability, preparedness constraints, response efficacy, self-efficacy, and the outcome-node CPTs also showed comparatively large ranges.

These findings complement the scenario results. Experienced disruption severity was strongly associated with variation in both preparedness outcomes, whereas repeated low-disruption experiences were associated primarily with increased risk normalization. The sensitivity rankings indicate that local variation in CPT parameters involving disruption severity produced the largest changes in the two target probabilities. These results represent parameter sensitivity rather than causal effects.

## 5. Discussion

### 5.1. Disruption Severity and a Learning-Consistent Preparedness Profile

A learning-consistent preparedness profile was more strongly associated with disruption severity than with exposure frequency alone. When experience frequency was fixed at Limited, increasing severity from Low to Moderate and High corresponded to higher probabilities of high supplies and outage preparedness, rising from 16% to 32% and 42%, and high mobility and care continuity preparedness, increasing from 16% to 28% and 35%. These changes were accompanied by higher risk perception, preparedness knowledge, and response efficacy, while risk normalization approached zero under high-severity conditions.

This pattern supports the argument that disaster experience becomes behaviorally meaningful when it involves concrete consequences and reveals weaknesses in household arrangements. Physical and material consequences are associated with risk perception, while emotional experience may operate indirectly through worry (Bronfman et al., 2020). Evidence from Wenchuan earthquake survivors also shows that disaster severity can remain positively associated with preparedness years later, particularly among vulnerable households (Xu et al., 2024). The present findings extend this evidence by identifying a conditional probabilistic pattern linking experienced severity with cognitive appraisal, efficacy beliefs, and preparedness outcomes.

The results are also consistent with evidence that confidence in one’s capacity to act is associated with greater preparedness, while household vulnerability shapes response capacity (Rao et al., 2022). General awareness of icy roads may be insufficient for action, whereas prolonged power or heating loss, restricted access to medicine, and disrupted care responsibilities provide more concrete evidence of household-continuity threats.

The Limited–High and Repeated–High comparison further qualifies this interpretation: the latter corresponded to greater preparedness knowledge, but both high-severity scenarios showed identical probabilities for the two high-preparedness outcomes; therefore, additional exposure may have diminishing returns once hazard seriousness is recognized, especially when financial, spatial, housing, time, and caregiving constraints limit action. Sensitivity analysis was consistent with this interpretation: parameters related to disruption severity produced the largest changes in A11 and A12 posteriors, while efficacy, vulnerability, and constraints were also influential. Severe experiences should therefore be interpreted as associated with, rather than automatically producing, preparedness.

### 5.2. Repeated Low-Disruption Experience and a Risk-Normalization Profile

The second major finding concerns repeated low-consequence experiences. Under low disruption severity, the probability of high risk normalization increased from 1% in None–Low to 17% in Limited–Low and 75% in Repeated–Low. In the latter scenario, high risk perception and response efficacy fell to 1% and 4%, while high supplies and outage preparedness and high mobility and care continuity preparedness were only 12% and 13%, respectively; thus, the dominant pattern was increased risk normalization rather than a large preparedness decline.

This pattern should not be equated with simple complacency. People facing tornado threats may actively assess changing risks while remaining constrained by vulnerability and limited response efficacy (Demuth et al., 2022). Similarly, households may understand freezing-rain warnings and available preparedness measures but infer from repeated minor impacts that additional action is unnecessary. Under Repeated–Low, preparedness knowledge remained more likely than high risk perception or response efficacy, suggesting that households may know what to do without perceiving sufficient reason to act.

Near-miss research offers a related interpretation. Flood-risk experiments show that near misses may reduce risk perception and mitigation intentions (Bogani et al., 2023), and evidence after Hurricane Irma indicates that a close call may lower self-efficacy while increasing personal responsibility for preparedness (Billman et al., 2023). Perceived warning-system inaccuracy can also weaken institutional credibility and future compliance (Ripberger et al., 2015). In freezing-rain events, limited impacts may therefore be attributed to low hazard severity rather than favorable timing, effective infrastructure, assistance, or chance.

The Repeated–Low and Repeated–High contrast shows that normalization is conditional rather than inevitable. Repeated severe disruption was associated with stronger hazard recognition and preparedness knowledge, whereas repeated minor disruption corresponded to expectations of limited consequences. This contrast may also reflect a broader sociocultural context: shared winter narratives, collective memories of service disruption, and social capital may shape whether recurrent freezing rain is framed as routine or consequential. However, these mechanisms were not directly measured, and A4 and A12 provide only limited functional proxies.

These findings suggest that communication after low-impact events should calibrate rather than heighten risk perceptions. Effective household communication requires phase-sensitive organizational coordination (Liu et al., 2024), and incentive or supervision arrangements may help translate warning information into local action under information asymmetry (Ma et al., 2024). Messages should explain why previous consequences remained limited, clarify how similar weather could become more disruptive, and provide feasible preparedness actions. Community staff, volunteers, and trusted local intermediaries can adapt this guidance while balancing risk awareness, emotional stability, and perceived capacity to act.

### 5.3. From Shared Drivers to Domain-Specific Preparedness

The third contribution concerns differences between the two preparedness outcomes. Repeated cross-validation showed stronger predictive discrimination for A11 than for A12: the former performed similarly to the multinomial logistic benchmark, whereas the latter showed limited-to-moderate discrimination, greater uncertainty, and weaker performance than the logistic benchmark, suggesting that preparedness varies not only in content but also in behavioral regularity.

Supplies and outage preparedness includes relatively standardized actions, such as storing food, water, medication, lighting, backup power, and communication resources. These measures can often be accumulated gradually and require limited coordination with external actors, making their associations with risk perception, knowledge, efficacy, and previous disruption comparatively stable. In contrast, mobility and care continuity preparedness are more context-dependent, involving travel adjustment, alternative transportation, emergency communication, temporary relocation, care for vulnerable household members, and access to community assistance. These actions depend on event timing, household composition, transport availability, social networks, and local service capacity. At the wider emergency-system level, modular coordination among heterogeneous organizations may support the external services on which household continuity plans depend (Liu et al., 2022; Liu & Dong, 2024).

The results extend evidence that different preparedness behaviors show distinct conditional profiles. Self-initiated and externally prompted emergency-material preparations have distinct motivational and perceptual determinants (Cao et al., 2023), and earthquake and tsunami research shows that hazard knowledge, risk perception, self-efficacy, and physical preparedness contribute differently to immediate and pre-evacuation intentions (Buylova et al., 2020). The present findings suggest that outage preparedness and mobility or care continuity should not be collapsed into one aggregate measure.

Preparedness implications should therefore be differentiated by behavioral domain, while A12-related recommendations should remain exploratory and context-dependent. Supply and outage preparedness can be supported through standardized checklists and seasonal reminders, whereas mobility and care continuity planning may require the individualized assessment of transport, caregiving, relocation, and nearby assistance. Community staff, volunteers, local service providers, and other trusted intermediaries may help households translate general guidance into feasible arrangements (Lu et al., 2026). These implications correspond to broader disaster-risk-reduction priorities, including calibrated risk communication, essential-service continuity, local risk management, and climate resilience, but do not imply universal applicability beyond the study setting.

**Figure 9:**
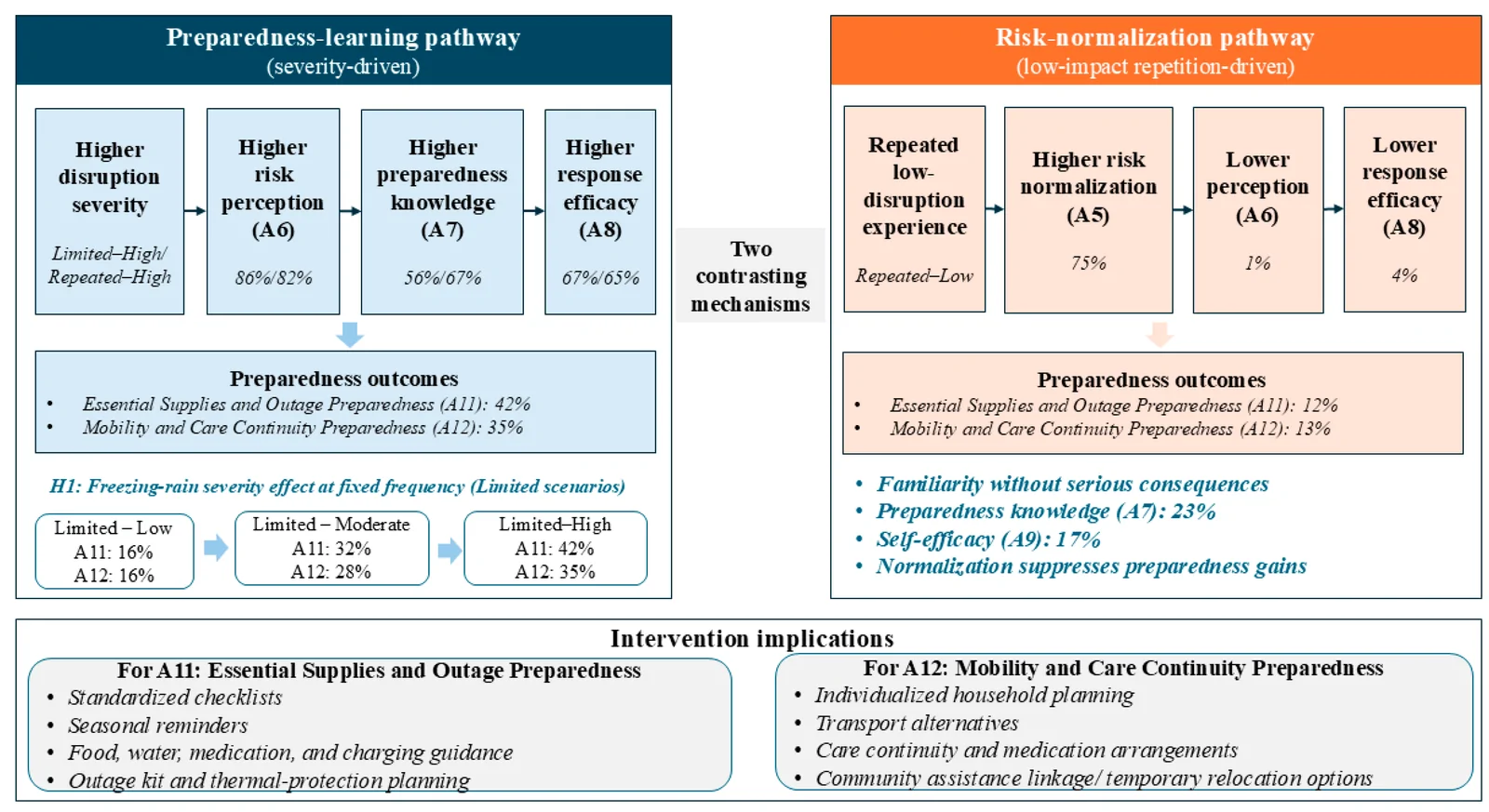
Contrasting conditional preparedness profiles and their policy implications. The profiles summarize conditional posterior associations and do not represent causal or temporal pathways.

Future models should incorporate transport dependence, caregiving intensity, social-support availability, neighborhood accessibility, and trust in community assistance. Emerging technologies may improve data analysis and interorganizational coordination under extreme disaster risk (Dong et al., 2026), while longitudinal data could distinguish households without continuity plans from those relying on flexible, event-contingent arrangements. Figure 9 summarizes the two conditional profiles and their policy implications.

## 6. Conclusions

In this study, a Bayesian network was used to examine how freezing-rain experience frequency and experienced disruption severity are associated with household risk interpretation and preparedness decisions. By distinguishing repeated exposure from reported disruption consequences, the analysis moved beyond the assumption that previous disaster experience has a uniformly positive association with preparedness. The model integrated risk normalization, risk perception, preparedness knowledge, response efficacy, self-efficacy, preparedness constraints, and two distinct preparedness outcomes within a single probabilistic framework.

The scenario results identified two contrasting conditional profiles. Increasing disruption severity was associated with higher probabilities of risk perception, preparedness knowledge, response efficacy, supplies and outage preparedness, and mobility and care continuity preparedness; in contrast, repeated low-disruption experience corresponded to substantially higher risk normalization and slightly lower probabilities of both preparedness outcomes. Repetition was associated with a normalization-consistent profile when previous impacts remained limited, whereas severe disruption was associated with a learning-consistent preparedness profile under both limited and repeated experiences. Although the H3 interaction contrasts were positive and statistically supported under the reported analyses, their substantive magnitudes were modest and were attenuated under the alternative discretization rules. They should therefore be interpreted as evidence of a modest conditional interaction pattern rather than a large or robustly strong interaction. These cross-sectional patterns should not be interpreted as causal effects or temporal learning processes.

The findings also indicate that household preparedness is not a unitary behavioral outcome. The Bayesian network showed stronger predictive performance for supplies and outage preparedness than for mobility and care continuity preparedness, suggesting that standardized material preparations were more readily explained by the modeled cognitive and experiential conditions. Mobility and care continuity arrangements appeared to be more dependent on household constraints, caregiving responsibilities, transport access, and external support, suggesting that preparedness guidance may need to combine general support for emergency supplies and outage continuity with more individualized planning for mobility, caregiving, temporary relocation, and community assistance. Such guidance should be communicated through trusted local intermediaries in a calibrated and action-oriented manner that supports emotional stability and perceived capacity to act.

Several limitations should be acknowledged: the cross-sectional design supports conditional probabilistic inference but does not establish causal relationships or temporal learning processes; self-reported experience and preparedness may involve recall and reporting biases; the five cities were purposively selected, recruitment used community-based and online convenience channels, and participation was subject to self-selection; the sample was also relatively highly educated, and one adult respondent represented each household. Consequently, the findings should not be generalized to all households in the study cities or to all freezing-rain-prone regions in China. Future research should use probability-based or longitudinal designs, incorporate social support and service-accessibility indicators, and validate the model across regions and winter hazards.

## Figures and Tables

**Table 1:** Measurement and definition of Bayesian network nodes.

Symbol	Layer	Node	Measurement	States
A1	Experience and household conditions	Freezing-Rain Experience Frequency	Number of directly experienced events during the previous five years	None: 0; Limited: 1–2; Repeated: ≥3
A2	Experience and household conditions	Experienced Disruption Severity	Maximum household impact involving mobility, services, health, or property	Low/Moderate/High
A3	Experience and household conditions	Warning Information Exposure	Four-item Likert scale on warning and preparedness information exposure	Low/Moderate/High
A4	Experience and household conditions	Household Disruption Vulnerability	Number of vulnerable members and service-dependence conditions	Low/Moderate/High
A5	Cognitive interpretation and learning	Freezing-Rain Risk Normalization	Five-item Likert scale	Low/Moderate/High
A6	Cognitive interpretation and learning	Household Disruption Risk Perception	Five-item Likert scale on likelihood and severity	Low/Moderate/High
A7	Cognitive interpretation and learning	Freezing-Rain Preparedness Knowledge	Five-item Likert scale	Low/Moderate/High
A8	Action appraisal and conditions	Preparedness Response Efficacy	Four-item Likert scale	Low/Moderate/High
A9	Action appraisal and conditions	Preparedness Self-Efficacy	Four-item Likert scale	Low/Moderate/High
A10	Action appraisal and conditions	Preparedness Constraints	Five-item Likert scale	Low/Moderate/High
A11	Household preparedness decisions	Essential Supplies and Outage Preparedness	Checklist of 10 completed actions	Low: 0–3; Moderate: 4–6; High: 7–10
A12	Household preparedness decisions	Mobility and Care Continuity Preparedness	Checklist of 8 completed actions	Low: 0–2; Moderate: 3–5; High: 6–8

Note: For A3 and A5–A10, Low = 1.00–2.33, Moderate = 2.34–3.67, and High = 3.68–5.00. For A4, Low = 0–1, Moderate = 2–3, and High = 4–6.

**Table 2:** Bayesian scenarios for hypothesis assessment.

	Scenario	Evidence	Main Posterior Outcomes	Assessment Criterion
H1	Increasing disruption severity	Experience frequency = limited; severity = low, moderate, and high	Two preparedness outcome nodes	Probability of high preparedness increases with severity
H2	Repeated low-disruption experience	Severity = low; frequency = limited and repeated	Risk normalization and two preparedness outcomes	Repeated–Low corresponds to higher normalization and lower preparedness
H3	Joint experience patterns	Limited/repeated frequency combined with low/high severity	Normalization, risk perception, knowledge, and preparedness	Posterior preparedness differs across the four configurations

**Table 3:** Characteristics of the analytical sample (N = 533).

Characteristic	Category	N	%
Province	Hunan	321	60.2
	Hubei	212	39.8
City	Changsha	148	27.8
	Yueyang	107	20.1
	Huaihua	66	12.4
	Wuhan	130	24.4
	Yichang	82	15.4
Gender	Male	253	47.5
	Female	280	52.5
Age Group	18–29	130	24.4
	30–39	142	26.6
	40–49	122	22.9
	50–59	102	19.1
	≥60	37	6.9
Education	High school or below	91	17.1
	College	142	26.6
	Bachelor	218	40.9
	Postgraduate	82	15.4
Monthly household income	<RMB 5000	195	36.6
	RMB 5000–9999	174	32.6
	RMB 10,000–14,999	94	17.6
	RMB 15,000–24,999	58	10.9
	≥RMB 25,000	12	2.3
Housing type	Owned	377	70.7
	Rented	136	25.5
	Other	20	3.8
Older adult in household	Yes	143	26.8
Young child in household	Yes	254	47.7
Household member with chronic illness	Yes	130	24.4
Dependence on electrically powered medical equipment	Yes	38	7.1
Limited access to transportation	Yes	121	22.7
Limited access to external assistance	Yes	94	17.6

**Table 4:** Measurement model assessment.

Construct	Items	Standardized Loading Range	Cronbach’s α	Composite Reliability	Average Variance Extracted
Warning Information Exposure	4	0.792–0.843	0.888	0.888	0.667
Freezing-Rain Risk Normalization	5	0.755–0.802	0.883	0.883	0.602
Household Disruption Risk Perception	5	0.840–0.862	0.929	0.929	0.724
Freezing-Rain Preparedness Knowledge	5	0.791–0.831	0.905	0.906	0.657
Preparedness Response Efficacy	4	0.724–0.746	0.824	0.824	0.539
Preparedness Self-Efficacy	4	0.729–0.779	0.838	0.838	0.565
Freezing-Rain Preparedness Constraints	5	0.733–0.770	0.867	0.867	0.566

**Table 5:** Posterior probabilities under the Bayesian scenarios.

Scenario	P (A5 High)	P (A6 High)	P (A7 High)	P (A8 High)	P (A9 High)	P (A11 High)	P (A12 High)
None–Low	1%	12%	21%	25%	15%	22%	21%
Limited–Low	17%	6%	14%	12%	14%	16%	16%
Limited–Moderate	1%	46%	44%	45%	22%	32%	28%
Limited–High	0%	86%	56%	67%	24%	42%	35%
Repeated–Low	75%	1%	23%	4%	17%	12%	13%
Repeated–High	0%	82%	67%	65%	27%	42%	35%

Note: A5 = Risk Normalization; A6 = Risk Perception; A7 = Preparedness Knowledge; A8 = Response Efficacy; A9 = Self-Efficacy; A11 = Supplies and Outage Preparedness; A12 = Mobility and Care Continuity Preparedness. All values represent the posterior probability that the corresponding node is in its High state.

**Table 6:** Bootstrap assessment of the hypotheses.

Hypothesis	Posterior Probability Contrast	Full Sample	Bootstrap Mean	SE	95% Percentile CI	Assessment
H1	A11: Limited–Moderate − Limited–Low	16.8	16.7	2.3	[12.5, 21.3]	Supported
	A11: Limited–High − Limited–Moderate	9.6	9.6	1.9	[6.1, 13.6]	
	A12: Limited–Moderate − Limited–Low	12.2	12.1	1.9	[8.6, 16.0]	
	A12: Limited–High − Limited–Moderate	6.5	6.5	1.5	[3.8, 9.6]	
H2	A5: Repeated–Low − Limited–Low	58.4	58.4	5.8	[47.2, 70.0]	Supported
	A11: Repeated–Low − Limited–Low	−4.1	−4.0	1.3	[−6.7, −1.7]	
	A12: Repeated–Low − Limited–Low	−3.2	−3.1	1.2	[−5.6, −0.8]	
H3	A11: Difference-in-differences	3.8	3.7	1.8	[0.2, 7.5]	Supported
	A12: Difference-in-differences	3.2	3.2	1.5	[0.4, 6.1]	

Note: Values are percentage-point differences based on unrounded posterior probabilities. SE = bootstrap standard error. Confidence intervals are percentile intervals obtained from 2000 nonparametric case-resampling bootstrap samples. For H3, the difference-in-differences was calculated as (Repeated–High − Repeated–Low) − (Limited–High − Limited–Low).

**Table 7:** Repeated cross-validation and benchmark comparison.

Outcome	Model	Accuracy	Balanced Accuracy	Macro-F1	AUC—Low	AUC—Moderate	AUC—High	Macro-AUC
A11	Bayesian network	0.650 ± 0.066	0.650 ± 0.069	0.648 ± 0.067	0.872 ± 0.051	0.692 ± 0.070	0.839 ± 0.063	0.801 ± 0.044
	Multinomial logistic	0.654 ± 0.064	0.653 ± 0.070	0.653 ± 0.066	0.881 ± 0.047	0.697 ± 0.069	0.854 ± 0.057	0.811 ± 0.042
	Majority class	0.478 ± 0.007	0.333 ± 0.000	0.216 ± 0.002	0.500 ± 0.000	0.500 ± 0.000	0.500 ± 0.000	0.500 ± 0.000
A12	Bayesian network	0.519 ± 0.066	0.480 ± 0.070	0.486 ± 0.076	0.766 ± 0.069	0.564 ± 0.082	0.763 ± 0.064	0.698 ± 0.053
	Multinomial logistic	0.552 ± 0.073	0.516 ± 0.077	0.524 ± 0.082	0.800 ± 0.062	0.605 ± 0.079	0.786 ± 0.060	0.730 ± 0.049
	Majority class	0.462 ± 0.008	0.333 ± 0.000	0.211 ± 0.002	0.500 ± 0.000	0.500 ± 0.000	0.500 ± 0.000	0.500 ± 0.000

Note: Values are means ± standard deviations across 100 test folds from 10 repetitions of stratified 10-fold cross-validation. A11 = Supplies and Outage Preparedness; A12 = Mobility and Care Continuity Preparedness. The Bayesian network and multinomial logistic benchmark used the same categorical predictors: A8, A9, and A10. AUC values were calculated using one-versus-rest comparisons.

## Data Availability

Data are available from the corresponding author upon reasonable request. The data are not publicly available because the individual-level questionnaire dataset contains combinations of geographic, demographic, disaster-experience, and household-preparedness variables that could create a risk of indirect participant identification.

## Supplementary Materials

The following supporting information can be downloaded at: https://www.mdpi.com/article/10.3390/bs16091533/s1, Table S1: Measurement properties of the risk-normalization scale; Table S2: Summary of standardized factor loadings; Table S3: Comparison of CFA models; Table S4: HTMT discriminant validity; Table S5: Joint distribution of A1 and A2; Table S6: CPT coverage and robustness diagnostics.
