# Supplementary material for: Freezing-Rain Experience and Household Preparedness: A Bayesian Network Analysis of Disruption Severity and Risk Normalization
Source: Behav Sci (Basel). 2026 Aug 31;16(9):1533. doi: 10.3390/bs16091533 (PMC13603182; doi:10.3390/bs16091533)
Supplement: Supplementary file 1 [file behavsci-16-01533-s001.zip › behavsci-4498484-supplementary.pdf]

Supplementary Materials: Table S1, Measurement properties of the risk-normalization scale; Table S2, Summary of standardized factor loadings; Table S3, Comparison of CFA models; Table S4, HTMT discriminant validity; Table S5, Joint distribution of A1 and A2; Table S6, CPT coverage and robustness diagnostics.

Supplementary Table S1. Measurement properties of the risk-normalization scale.

Item	Item wording	Mean	SD	Standardized loading
RN1	Previous freezing-rain events caused little harm to my household.	2.657	1.279	0.755
RN2	Freezing-rain warnings are usually more serious than the actual consequences.	2.625	1.232	0.773
RN3	My household can manage freezing rain without special advance preparation.	2.698	1.311	0.774
RN4	Because past events were minor, future freezing rain is unlikely to be serious.	2.589	1.259	0.777
RN5	Freezing rain is mainly a routine winter inconvenience.	2.612	1.204	0.802

Note: N = 533. All items were measured using a five-point Likert scale ranging from 1 (strongly disagree) to 5 (strongly agree), with higher scores indicating stronger risk normalization. The scale was developed for this study based on near-miss interpretation and risk-normalization research, including that of Dillon et al. (2014) and Tinsley et al. (2012). The initial items were reviewed by an eight-member expert panel and refined through pilot testing before the formal survey. Confirmatory factor analysis supported the five-item structure: Cronbach's α = 0.883, composite reliability = 0.883, and average variance extracted = 0.602. All standardized loadings exceeded 0.70.

Supplementary Table S2. Summary of standardized factor loadings.

Construct	Items	Loading range	Mean loading
Warning Information Exposure	4	0.792–0.843	0.816
Freezing-Rain Risk Normalization	5	0.755–0.802	0.776
Household Disruption Risk Perception	5	0.840–0.862	0.851
Freezing-Rain Preparedness Knowledge	5	0.791–0.831	0.811
Preparedness Response Efficacy	4	0.724–0.746	0.734
Preparedness Self-Efficacy	4	0.729–0.779	0.751
Freezing-Rain Preparedness Constraints	5	0.733–0.770	0.752

Note: Standardized loadings were estimated from the seven-factor confirmatory measurement model using the 32 original five-point Likert items. All item loadings exceeded 0.70.

Supplementary Table S3. Comparison of CFA models.

Model	χ^2	df	χ^2/df	CFI	TLI	RMSEA	RMSEA 90% CI	SRMR
Seven-factor model	415.478	443	0.938	1.000	1.003	0.000	0.000–0.009	0.026
RN and RP merged	2053.679	449	4.574	0.830	0.812	0.082	0.081-0.083	0.161
RN and RE merged	1456.452	449	3.244	0.893	0.882	0.065	0.063-0.066	0.140
One-factor model	7099.117	464	15.300	0.298	0.250	0.164	0.163-0.165	0.230

Note: The seven-factor model specified warning information exposure, freezing-rain risk normalization, household disruption risk perception, freezing-rain preparedness knowledge, preparedness response efficacy, preparedness self-efficacy, and freezing-rain preparedness constraints as seven correlated reflective factors. The first six-factor model combined risk normalization and risk perception, while the second combined risk normalization and response efficacy. The one-factor model loaded all 32 items on a single general factor. RN = risk normalization; RP = risk perception; RE = response efficacy; CFI = comparative fit index; TLI = Tucker–Lewis index; RMSEA = root mean square error of approximation; SRMR = standardized root mean square residual.

Supplementary Table S4. HTMT discriminant validity.

Panel A. HTMT matrix.

Construct	WIE	RN	RP	PK	RE	SE	PC
WIE	-						
RN	0.070	-					
RP	0.060	0.679	-				
PK	0.194	0.272	0.257	-			
RE	0.027	0.676	0.675	0.374	-		
SE	0.102	0.118	0.056	0.280	0.114	-	
PC	0.044	0.034	0.135	0.043	0.100	0.417	-

Panel B. Bootstrap confidence intervals for the largest HTMT values.

Construct pair	HTMT	Bootstrap 95% CI
RN–RP	0.679	0.618–0.731
RN–RE	0.676	0.618–0.730
RP–RE	0.675	0.610–0.736

Note: HTMT = heterotrait–monotrait ratio of correlations. Confidence intervals were obtained from 5000 nonparametric bootstrap samples. All HTMT values were below 0.85, and the bootstrap confidence intervals of the three largest HTMT values remained below 1.00. WIE = Warning Information Exposure; RN = Freezing-Rain Risk Normalization; RP = Household Disruption Risk Perception; PK = Freezing-Rain Preparedness Knowledge; RE = Preparedness Response Efficacy; SE = Preparedness Self-Efficacy; PC = Freezing-Rain Preparedness Constraints.

Supplementary Table S5. Joint distribution of A1 and A2.

Experience frequency	Low	Moderate	High	Total
None	26 (4.9%)	16 (3.0%)	20 (3.8%)	62 (11.6%)
Limited	85 (15.9%)	47 (8.8%)	78 (14.6%)	210 (39.4%)
Repeated	102 (19.1%)	75 (14.1%)	84 (15.8%)	261 (49.0%)
Total	213 (40.0%)	138 (25.9%)	182 (34.1%)	533 (100.0%)

Note: Percentages refer to the full sample. All nine A1 × A2 configurations were observed.

Supplementary Table S6. CPT coverage and robustness diagnostics.

Panel A. CPT coverage by node.

Node	Parents	Parent-state rows	Minimum N	Median N	N=0 rows	N<5 rows	N<10 rows
A1	None	1	533	533	0	0	0
A2	None	1	533	533	0	0	0
A3	None	1	533	533	0	0	0
A4	None	1	533	533	0	0	0
A5	A1, A2	9	16	75	0	0	0
A6	A2, A4, A5	27	0	13	4	8	8
A7	A1, A2, A3	27	3	18	0	1	9
A8	A5, A6, A7	27	0	12	3	8	12
A9	A7, A10	9	10	56	0	0	0
A10	A4	3	107	176	0	0	0
A11	A8, A9, A10	27	1	21	0	5	7
A12	A8, A9, A10	27	1	21	0	5	7
Total	—	160	—	—	7	27	43

Note: Parent-state rows include the four root-node rows. Counts were calculated from the 533-case analytical sample before smoothing. The primary symmetric Dirichlet estimator assigned positive probability to all child states, including the seven unobserved parent-state rows.

Panel B. Robustness of the principal scenario contrasts.

Hypothesis	Posterior probability contrast	Primary model	Unsmoothed CPT	Empirical tertiles	Stricter High	Direction consistent
H1	A11: Limited–Moderate − Limited–Low	16.8	19.2	13.1	12.3	Yes
	A11: Limited–High − Limited–Moderate	9.6	10.2	5.0	4.9	Yes
	A12: Limited–Moderate − Limited–Low	12.2	13.8	10.1	9.7	Yes
	A12: Limited–High − Limited–Moderate	6.5	6.9	4.0	3.9	Yes
H2	A5: Repeated–Low − Limited–Low	58.4	59.0	48.4	33.0	Yes
	A11: Repeated–Low − Limited–Low	-4.1	-4.4	-2.5	-2.4	Yes
	A12: Repeated–Low − Limited–Low	-3.2	-3.5	-2.0	-1.9	Yes
H3	A11: Difference-in-differences	3.8	4.2	2.0	1.8	Yes
	A12: Difference-in-differences	3.2	3.6	1.6	1.4	Yes

Note: Values are full-sample posterior probability differences in percentage points. The primary model used the prespecified discretization rules and symmetric Dirichlet CPT estimation with α=1/3 for each child state. The unsmoothed specification used maximum-likelihood estimates for observed parent-state rows; seven unobserved rows were assigned uniform probabilities of 1/3 solely to keep

the network fully defined. The empirical-tertile and stricter-High specifications retained the primary Dirichlet estimator but changed the discretization rules. For H3, the difference-in-differences was calculated as (Repeated–High − Repeated–Low) − (Limited–High − Limited–Low).
